# Neutral buoyancy and the static perception of upright

**DOI:** 10.1038/s41526-023-00296-x

**Published:** 2023-06-28

**Authors:** Heather Jenkin, Michael Jenkin, Laurence R. Harris, Rainer Herpers

**Affiliations:** 1 Department of Psychology, York University, Toronto, Canada; 2 Centre for Vision Research, York University, Toronto, Canada; 3 Department of Electrical Engineering and Computer Science, York University, Toronto, Canada; 4 Institute of Visual Computing, Bonn-Rhein-Sieg University of Applied Sciences, St Augustin, Germany

**Keywords:** Human behaviour, Electrical and electronic engineering

## Abstract

The perceptual upright results from the multisensory integration of the directions indicated by vision and gravity as well as a prior assumption that upright is towards the head. The direction of gravity is signalled by multiple cues, the predominant of which are the otoliths of the vestibular system and somatosensory information from contact with the support surface. Here, we used neutral buoyancy to remove somatosensory information while retaining vestibular cues, thus “splitting the gravity vector” leaving only the vestibular component. In this way, neutral buoyancy can be used as a microgravity analogue. We assessed spatial orientation using the oriented character recognition test (OChaRT, which yields the perceptual upright, PU) under both neutrally buoyant and terrestrial conditions. The effect of visual cues to upright (the visual effect) was reduced under neutral buoyancy compared to on land but the influence of gravity was unaffected. We found no significant change in the relative weighting of vision, gravity, or body cues, in contrast to results found both in long-duration microgravity and during head-down bed rest. These results indicate a relatively minor role for somatosensation in determining the perceptual upright in the presence of vestibular cues. Short-duration neutral buoyancy is a weak analogue for microgravity exposure in terms of its perceptual consequences compared to long-duration head-down bed rest.

## Introduction

The perceptual upright results from the multisensory integration of the directions indicated by vision and gravity as well as the prior assumption that upright is towards the head. The direction of gravity is signalled by multiple cues, including the otoliths of the vestibular system and somatosensory information from contact with support surfaces. The relative importance of these cues can be separated using neutral buoyancy which essentially removes the somatosensory cue and produces a sensation of being weightless: as such, neutral buoyancy has been extensively and routinely used as an environment for training astronauts. Note, however, that this separation of vestibular and somatosensory cues is not complete because of low-level neural pathways connecting the proprioceptive and vestibular systems^1^. Neutral buoyancy environments permit six-degrees-of-freedom motions to be practiced and experiments to be performed with full-scale mock-ups of space hardware. But, as with other space analogue environments on Earth, the neutral buoyancy environment is not a perfect analogue for microgravity. Properly weighted objects may float in neutral buoyancy, but water is not a vacuum and the resistance of the water column, the inertia of water and its impact on objects do not match the effects experienced in air in a space station or in the vacuum of space during extravehicular activities.

Neutral buoyancy may impact human perception and performance in a way that may or may not match either Earth-normal behaviour, behaviour in microgravity, or the behaviour found in space analogues such as human centrifugation^2^, microgravity aircraft flight^3–6^ or long-duration bed rest^7^. Neutral buoyancy also introduces its own complications including distortions in visual perception due to light refraction at the glass-water interface between the participant’s goggles and the water column, and the effects of water pressure on the ears, an effect that is known to contribute to diver disorientation (see^8^ and^9^ for reviews). Does neutral buoyancy interfere with perceptual systems? If so, are any such influences similar to those found in spaceflight?

In space, both somatosensory and vestibular cues to the direction of gravity are compromised but in neutral buoyancy the vestibular cue remains unaffected while only the somatosensory cue, normally provided by pressure at the support surface, is disabled^10,11^, but see^8^ for the potential of low-level connections between the vestibular and proprioceptive systems which argues for a low-level interaction between the two systems. Here we consider whether lack of the somatosensory cue affects the perception of self-orientation under neutral buoyancy conditions.

Several studies that have explored the effect of water immersion on balance and the perception of self-orientation. For example, Glass and colleagues^8^ investigated the impact of long-term immersion on balance upon return to a non-buoyant environment, and others have compared the perception of self-orientation under neutral buoyancy conditions with responses under terrestrial, Earth-normal conditions^11,12^, and^13^. Traditionally, self-orientation experiments have evaluated this perception using the luminous line (LL) test to measure the subjective visual vertical (SVV). In this test participants adjust the orientation of a line until it is perceived to be aligned with gravity. Wade^11^ summarized rather succinctly the effect of buoyancy on the SVV observing that visual orientation constancy is only marginally reduced by immersion in water. The LL test suffers from important limitations. First, the LL test cannot be used when there is no gravity direction with which to align the line. Second, while the SVV is influenced by factors other than gravity, these factors play a relatively small role^14,15^. The SVV is therefore a relatively insensitive measure of the multisensory determinants of self-orientation. Third, the SVV introduces cognitive factors, including cueing the participant about the specific purpose of the task because it requires participants to consciously examine their perceived “up” direction.

Given these limitations of the LL test, we developed an alternative measure of perceived self-orientation known as the Oriented Character Recognition Test (OChaRT)^15^ for use in microgravity^16^. This test identifies the orientation at which an ambiguous character (e.g., the letter “p”) whose interpretation depends on its orientation (e.g., either a “d” or a “p”) appears least ambiguous. Since the participant’s task is to identify the character, there is no need to make a cognitive comparison with gravity. We refer to the orientation that this test yields as the perceptual upright (PU). By systematically varying the orientation of the visual or body cues to upright relative to gravity by viewing the character against a tilted background and by positioning the participant on their side, the relative contributions of each of these cues relative to gravity can be ascertained. Furthermore, visual cues to orientation can be removed by displaying the character against a featureless background, and the influence of gravity can be removed from the plane of testing by lying supine. By using such manipulations, it has been determined that the PU is more evenly influenced by the contributing cues than is the SVV. A typical distribution of the relative contributions of the components is 54% body, 25% vision and 21% gravity for the PU, compared to 15% body, 8% vision and 77% body for the SVV^15^. The weights G:1.0 V:1.2 and B:2.6 given in ref. ^15^ relative to “G” can be converted to percentages by using, for example for vision, V = 100% * (1.2/(1 + 1.2 + 2.6)) = 25%.

OChaRT has been used to study the perception of self-orientation under a range of different experimental conditions including body orientation relative to gravity^17^, while undergoing human centrifugation to explore human performance under different gravitational loads^2^, during short-duration microgravity generated in parabolic flight^18^, and during head-down bed rest (HDBR)^7^. Using OChaRT, Harris and colleagues^17^ demonstrated systematic changes in the perception of the PU during and following long-duration spaceflight. They reported that the ratio of the weightings of visual cues relative to body cues that determine the PU decreased when tested early during spaceflight. This effect disappeared later in flight but re-appeared a few weeks after return to a 1 G environment. If neutral buoyancy were a suitable microgravity analogue for the perception of self-orientation, then it should elicit similar effects. Jenkin^19^ conducted a preliminary study on the effect of buoyancy and although the small participant pool prevented a rigorous analysis of the data, results suggested that neutral buoyancy could influence the perceptual up as captured by OChaRT. Given the potential for neutral buoyancy to act as an inexpensive analogue for long-duration microgravity we therefore performed OChaRT for both ‘dry’ and ‘wet’ (buoyant) conditions to investigate if the observed reduction in visual weighting associated with long-duration microgravity exposure were also found when neutrally buoyant.

## Results

### Influence of body orientation

**Fig. 1:**
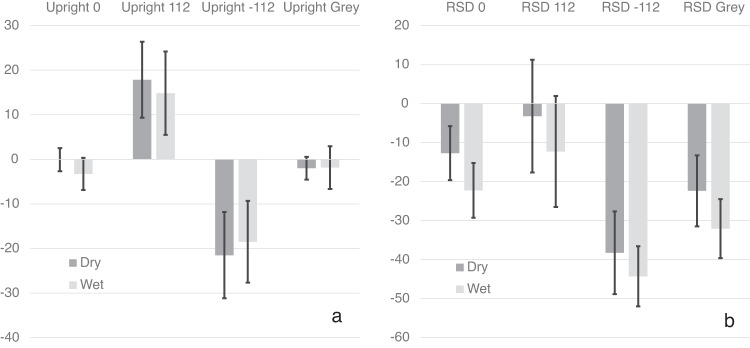
The effect of body posture and visual cues aligned with gravity on the PU. Data are plotted for upright (**a**) and right side down (**b**) body postures. The vertical axis shows angle in degrees where 0° corresponds to “PU aligned with the body” and positive numbers corresponds to “to the right of the body”. Standard error bars are shown.

Figure 1 shows the influence of body orientation and visual cue orientation on the mean PU plotted relative to the body. Repeated measures analyses on the PU were run separately for upright and right side down (RSD) participants. The full statistical analysis is given in Tables 1 (Upright) and 2 (RSD).

**Table 1:** PU analysis for upright participants.

Predictor	df_Num_	df_Dem_	F	*p*	ε_p_^2^
Buoyancy	1	9	0.12	0.740	0.013
Background	1.016	9.145	4.48	0.062	0.333
Buoyancy × Background	3	27	3.00	0.048	0.250

### Influence of buoyancy

**Fig. 2:**
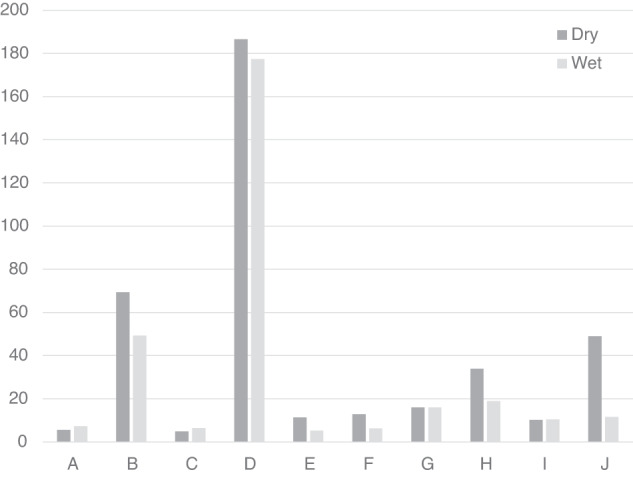
The effect of vision on the PU while upright plotted by participant as captured by the observer’s visual effect (VE). The difference between the PU when vision was orientated to the left and right is defined as the visual effect since the only difference in these conditions is the direction of the visual cue. The vertical axis is VE in degrees, while the horizontal axis shows each participant. Note the considerable variability of individual participant performance.

There was a significant interaction between buoyancy and background for upright participants. That an interaction rather than a main effect is found is not surprising as the upright and grey conditions are unlikely to see any difference with change in buoyancy given the alignment of the gravity and body vectors with the visual input for visual orientation of 0°. The interaction effect was explored through an analysis of the effect of vision on the PU for upright participants. The difference in PU caused by altering the visual background (here between +112° and −112°) reveals the effect of vision on the perceived direction of upright while keeping the other cues constant and is referred to as the visual effect (VE). Figure 2 plots the visual effect (VE) for each upright participant in both dry and wet conditions. A two-tailed t-test on VE shows a significant effect of buoyancy t(9) = −2.33, *p* = 0.045 in reducing the VE.

**Table 2:** PU analysis for RSD participants.

Predictor	df_Num_	df_Dem_	F	*p*	ε_p_^2^
Buoyancy	1	9	0.848	0.381	0.086
Background	1.042	9.378	3.206	0.105	0.263
Buoyancy × Background	3	27	0.329	0.805	0.035

### Gravity effect

**Fig. 3:**
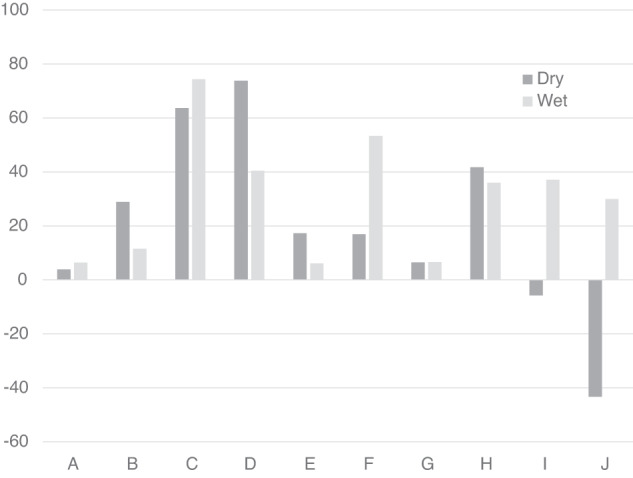
The effect of gravity on the PU plotted by participant as captured by the observer’s gravity effect (GE). The vertical axis is GE in degrees, while the horizontal axis shows each participant. There was no significant effect of buoyancy.

The gravity effect (GE) is defined as the difference in OChaRT performance between upright grey and right side down grey responses. This represents the difference in response when the body is rotated 90 degrees relative to gravity while observing a featureless background. Figure 3 plots the GE for each participant in dry and wet conditions. A two-tailed t-test on the GE shows no significant effect of buoyancy value of t(9) = 0.885, *p* = 0.398.

### The weighted vector sum model

**Fig. 4:**
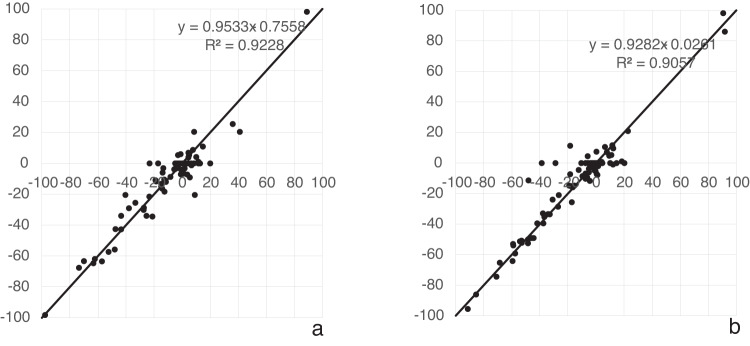
Assessing the linear weighted vector sum model. The weighted vector sum model described, models the PU as a weighted linear vector sum of vectors aligned with gravity, the body, and the visual display. Here we plot the model’s prediction in degrees (vertical axis) against participant responses (horizontal axis) for the PU for dry (**a**) and wet (**b**) conditions. The plot has a slope of dry: 0.95 with a y-intercept of −0.76; wet: 0.93 with a y-intercept of −0.03. These fits are drawn as a dotted line. A perfect fit is drawn as a solid line.

The pattern of responses to variations in the orientation of the body and visual cues can be used to probe how the cues are combined to provide a perception of the direction of up. The PU can be modelled as a linear weighted sum of three vectors pointing in the directions signalled by visual, body and gravity cues as follows^15^:

1 $${\rm{up}}={\rm{vision}}* {{\rm{weight}}}_{{\rm{vision}}}+{\rm{body}}* {{\rm{weight}}}_{{\rm{body}}}+{\rm{gravity}}* {{\rm{weight}}}_{{\rm{gravity}}}$$

where vision, body, and gravity are unit vectors in the appropriate directions associated with each cue, each with its own weighting expressed relative to the others. The weighted vector sum model has proven sufficient to explain a number of cue integration results (e.g., refs. ^15,16^) although more sophisticated models exist (e.g., refs. ^14,20^). Our procedure separated the directions indicated by each cue so that the relative magnitudes of the weights could be calculated. The PU measured in the upright and right-side-down conditions (thus varying the direction of gravity relative to the body) and with different visual background orientations (thus varying the direction of the visual cues relative to the body) were fitted to Eq. 1 for the wet and dry conditions separately. The three-vector model was fitted using a non-linear least-squares optimization for each probe-body orientation condition using Python’s SciPy minimization function configured to use the Broyden–Fletcher–Goldfarb–Shanno algorithm. Figure 4 shows the effectiveness of the model in predicting the data. This procedure provided the relative weighting of vision, body and gravity cues contributing to the PU for the dry (Fig. 4a) and wet (Fig. 4b) conditions.

**Fig. 5:**
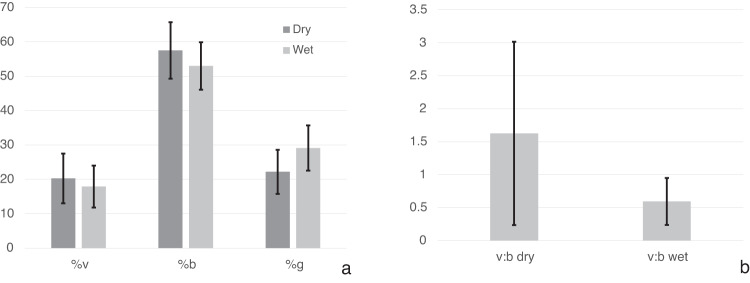
Modelling the PU using the weighted vector sum model. The relative weighting of the body (b), gravity (g) and visual (v) cues are shown for the PU for each measurement session (**a**). The ratio of vision-to-body weights for the dry and wet conditions (**b**).

The vision, body, and gravity weightings are compared for the dry and wet conditions in Fig. 5a. Differences were evaluated using two-tailed t-tests: %v t(9) = 1.5715, *p* = 0.151 n.s., %b t(9) = 0.7363, *p* = 0.4801 n.s., %g t(9) = −1.0558, *p* = 0.319 n.s. Following the procedure suggested by Harris and colleagues^16^, changes in relative weighting between dry and wet conditions were further examined using the ratio of the vision-to-body cue weights (Fig. 5b). The ratio of vision-to-body cues that determine the PU declined from dry to wet conditions, dropping from a ratio of 1.62 (dry) to 0.59 (wet). This decline was not statistically significant t(9) = 0.993, *p* = 0.347 n.s.

### Variance

Individual participant responses were fit with the product of two hyperbolic tangents (Eq. 2).

2 $${\rm{Fit}}({\rm{x;x}}0,{\rm{x}}1,{\rm{t}})=0.5* (1{{\mbox{-}}}{{\tanh }}(({\rm{x}}-{\rm{x}}0)/{\rm{t}})* {{\tanh }}(({\rm{x}}-{\rm{x}}1)/{\rm{t}}))$$

**Fig. 6:**
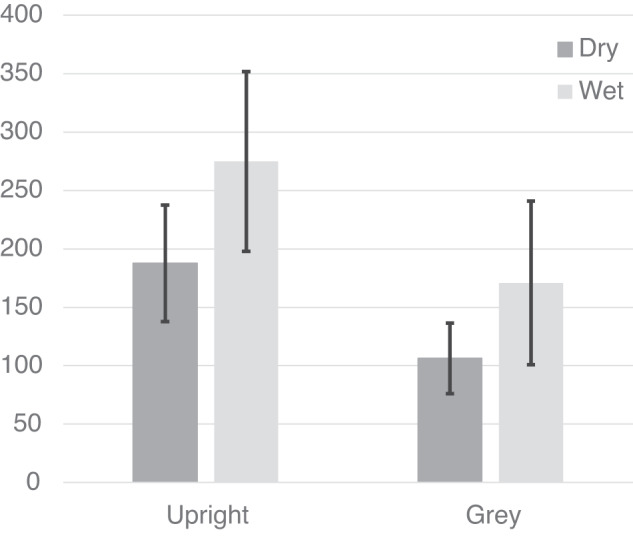
PU variances for conditions in which cues present were aligned. The vertical axis shows variance in degrees while the horizontal axis shows the different visual backgrounds. Data from both dry and wet conditions is shown. Standard errors are shown.

The t values in these equations were converted to degrees and used to identify the σ (standard deviations) for the best-fit Gaussian approximation to the hyperbolic tangent. The variance (σ^2^) of the PU estimates the variability associated with each of the cues. Of interest here is to compare the dry and wet conditions under which the body and gravity (and visual cues if present) agree. This corresponds to conditions when the participant is upright viewing an upright scene and when the participant is upright viewing a grey screen. The mean variances obtained under each of these conditions are plotted in Fig. 6. We might expect variance to decrease as more cues became available (upright scene visible) and to increase when the task is performed when underwater where factors such as cognitive loading, potential fogging of their SCUBA mask, etc., might impact participant performance. A repeated measures analysis of PU variances was conducted for session (2: dry versus wet) × cue (2: upright versus grey). There was no significant effect of buoyancy, background or interaction between buoyancy and background (Table 3).

**Table 3:** Analysis of PU variances.

Predictor	df_Num_	df_Dem_	F	*p*	ε_p_^2^
Buoyancy	1	9	2.811	0.128	0.238
Background	1	9	2.759	0.131	0.235
Buoyancy × Background	1	9	0.055	0.820	0.006

## Discussion

Although the use of visual cues may be less influential in determining the perceptual upright under neutral buoyancy conditions, we found no significant change associated with being underwater in the relative weighting of visual, body or gravity cues in this determination. Taking away the somatosensory component of the vestibulo-somatosensory cue to the direction of gravity by neutral buoyancy demonstrated no significant effect in the study, suggesting the dominance of the vestibular cue over somatosensory cues in determining perceptual upright. Surprisingly, there was no significant increase in the variance of orientation judgements underwater despite the known difficulties of orienting oneself underwater^12^. Previous studies have shown that the subjective visual vertical is also unaffected by neutral buoyancy^11–13^. Using the greater sensitivity of the Oriented Character Recognition Test (OChaRT) to the influence of the visual input on the perceptual upright (PU) we were able to demonstrate that neutral buoyancy does influence the perception of self-orientation and that buoyancy does impact the perception of self-orientation as captured by the visual effect (VE). This confirms Jenkin and colleagues’ preliminary study^19^ which suggested a shift in the PU as a consequence of neutral buoyancy.

Although a reduced contribution of vision in determining the PU was confirmed, the influence of neutral buoyancy on the PU was not reflected in the relative contributions of body, gravity and vision revealed by a linear weighted sum model. This is disappointing as both long-duration microgravity^16^ and head-down bed rest^7^ have shown a decline in the ratio of vision:body weightings. Harris and colleagues^16^ observed that the VE decreased upon initial exposure to long-duration microgravity (between 9 and 14 days after launch) and showed that this decrease was reflected in significant changes in the ratio of vision:body in terms of a linear weighted vector sum model of the perception of upright. They found a similar effect after 21 days of head-down bed rest^7^: the VE decreased and was reflected in a significant change in the vision:body ratio. The lack of a reduction in the vision:body weighting ratio in neutral buoyancy is a concern in terms of the use of neutral buoyancy as a perceptual analogue for long-duration microgravity.

One aspect of human performance illustrated here that replicates the results of earlier terrestrial experiments is the wide inter-subject variation in performance. As an analogue environment for microgravity, neutral buoyancy creates some concerns and has some limitations. The wide variability we noted in participant response is unlikely to be due exclusively to variations in the experimental environment such as water resistance and inertia, cognitive loading, goggle fogging, and inner ear trauma. All our participants were experienced scuba divers, and none reported any such environmental issues during post data collection debriefing. Rather, the variability more likely reflects inter-individual variation. Some of our participants could be identified as ‘gravity dominant’ in that they were highly influenced by the gravity vector, while others could be identified as ‘body dominant’ in that their responses were highly influenced by the direction of their body. This high variance in our participants may have obscured some significant changes. Further studies with a larger participant pool are needed.

Interestingly, gravity weighting was unaffected by neutral buoyancy. The direction of gravity is normally detected by the otoliths of the vestibular system and pressure on the support surface detected by the somatosensory system^10^. This lack of effect of submersion is surprising in light of the extensive interactions of vestibular and somatosensory information in the brain^21^ and in postural control^22,23^. The lack of an effect of removing somatosensory cues to orientation suggests that the PU is coded essentially in a head-centred reference frame in a normal gravity environment. If somatosensory cues are provided while in microgravity, they can then dominate orientation perception. For example, if pressure is applied to the top of the head of an astronaut on the International Space Station, the astronaut can have a sudden feeling of standing on their head^24^. Similarly, pressure applied to the soles of the feet can reverse this sensation^25^. However, it seems from our study that when present, even underwater, the vestibular signal dominates.

## Methods

### Participants

**Fig. 7:**
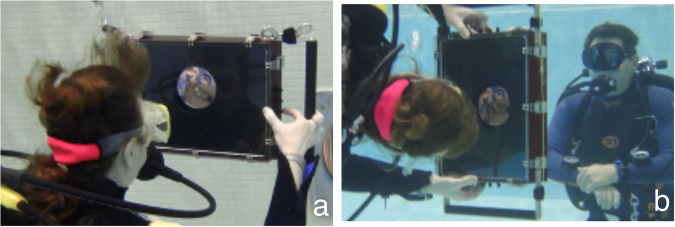
The body orientations and equipment used in this study. Stimuli were presented on a screen viewed within a custom underwater housing that contained the display and data collection computer. The participant viewed the stimuli either upright (**a**) or right side down (**b**). The housing was secured to lines running from weights on the bottom of the pool to lift bags. A safety diver was on site to deal with any possible emergency.

Ten participants (mean age 44 years, SD ± 9 years, range 23–51 years, 5 female) participated in the study, which was pre-approved by the ethic committees of York University and conformed to all applicable clauses of the Declaration of Helsinki^26^ which were relevant for our study except for clause #35. All participants had normal or corrected-to-normal vision. None reported any history of vestibular disease or damage. All participants were experienced SCUBA divers and were recruited from local SCUBA clubs and user groups. None had been a previous participant in experiments involving the visual stimuli used here. Each participant read and signed an informed consent statement before testing began. Participants received no compensation for their participation in the study. The authors affirm that human research participants provided informed consent for publication of the images in Fig. 7.

### Equipment

Participants were tested in two environmental conditions (in a dry laboratory setting and underwater at either the Tait Mackenzie Pool at York University Canada, or the Diving Tower at the Friendly Cityhotel Oktopus in Siegburg, Germany) and in two body postures (upright and right-side down).

Stimuli were displayed on a Lenovo X201 Tablet computer (1280 × 800 resolution) which was mounted within a 35.5 cm (width) × 24.1 cm (height) × 10.1 cm (depth) custom underwater housing with mechanical switches mounted on its external edges for input. The screen was viewed through a circular aperture (diameter 10 cm). Participants were instructed to hold the housing with their elbows at 90° resulting in a field of view of approximately 19° (see Fig. 7). The SCUBA mask they were wearing occluded peripheral vision.

Participants held on to the underwater housing through large handles mounted on the device which could be mounted either horizontally or vertically. The same display was used for both on surface (dry) and neutrally buoyant (wet) data collection sessions. For dry data collection sessions, the housing was mounted vertically against supports. For wet data collection the housing was secured to lines running from weights on the bottom of the pool to lift bags. A second rope from the surface, supported by another lift bag, was used to provide support for the participant’s legs when they were on their side. Wet data were collected with the participant neutrally buoyant with their head between 1 and 2 m below the surface of the water. For all data collection sessions participants wore their normal thermal protection (typically a wet suit) and their SCUBA mask. For wet data collection sessions, participants also wore the rest of their normal SCUBA gear including regulator, buoyancy compensation device (BCD), and fins (see Fig. 7).

### Stimuli

**Fig. 8:**
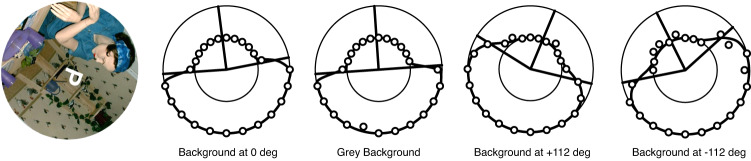
Sample responses. Responses for one participant for the four backgrounds for a single body orientation (here upright) and buoyancy condition (here wet). The figure in the visual display is a photograph of a manikin that provided realistic orientation cues. A product of two psychometric functions is plotted through the data in polar coordinates where the outer circle represents “probe interpreted as a ‘d’ 100% of the time” and the inner circle represents “probe interpreted as a ‘p’ 100% of the time”. The PU is defined as the midpoint between the PSEs (50% points) of the two psychometric functions, indicated by radial lines.

All probes were presented in front of a visual background that was either grey (no visual cues to upright) or a highly polarized scene with many visual cues to the direction of gravity that was displayed either upright or rotated ±112° relative to the body axis (Fig. 8). For each body orientation (upright and right side down), the probe was presented against these four backgrounds. The perceptual upright (PU) was measured by means of the oriented character recognition test (OChaRT)^15^. We used the ambiguous character “p” as the probe. The character appears as a “p” in one orientation and a “d” when rotated by 180°. The character measured 1.76° × 1.49° when viewed at the viewing distance of 21 cm. The probe was presented at one of 24 different orientations equally spaced around the roll axis and the participant’s task was to indicate if the probe appeared to be a “p” or a “d”. The orientations at which it appeared most ambiguous were assessed from which the perceptual upright, defined as the orientation midway between these most-ambiguous orientations, was calculated (see Fig. 8). The probe was presented for 500 ms, and the display was then replaced with a grey background and a circular fixation marker. The display was presented until the participant responded. The ambiguous letter probe was presented every 15° (24 equally spaced orientations) on each of the four backgrounds. Each probe was presented 7 times for a total of 24 × 7 × 4 = 672 trials per body orientation. Sample participant responses are illustrated in Fig. 8.

### Procedure

Participants were tested in two environmental conditions (‘dry’ in a laboratory and ‘wet’ underwater) and in two body postures (upright and right side down). The order in which a participant experienced the environmental conditions (wet and dry) was randomized but once in that environment, the participant completed all the in-pool or all the in-lab data collection sessions. The order of the body postures was randomly assigned and randomized within the in-lab and the in-pool groups.

### Data analysis

Positive angles are clockwise from the participant’s point of view. All data are described here in a body-centric coordinate system where the body midline is defined as 0°. The up direction is defined by gravity is therefore at −90° for the right-side down viewing conditions.

The orientation of the PU was determined by fitting a double psychometric function to the frequency with which participants chose “p”. The data were fit with a product of two hyperbolic tangents (Eq. 2) with a common scale factor (t). The PU was defined as halfway between the two points of maximum ambiguity (x0 and x1). t provides an estimate of the variability of the participant’s response. Sample results for the PU and SVV along with their fits are given in Fig. 8.

The data were analysed using SPSS v28. Repeated measures ANOVAs were used as the primary statistical test. Tests that violated sphericity had their degrees of freedom corrected using Greenhouse-Geisser when appropriate. Post hoc t-tests were performed using Bonferroni correction.

## Data Availability

OChaRT responses (PU values) by anonymized participants that support the findings of this study are available at Borealis. 10.5683/SP3/M9M6VY.
